# Proximity and current alignment drive fertilisation success in a broadcast-spawning coral (*Acropora* cf. *hyacinthus*)

**DOI:** 10.1038/s42003-026-10055-9

**Published:** 2026-05-09

**Authors:** Gerard F. Ricardo, Christopher Doropoulos, Adriana Humanes, Liam Lachs, Helios M. Martinez, Greta Sartori, Taison Ka Tai Chang, Apple Pui Yi Chui, Elvis Long Ching Wong, Jamie R. Stevens, Iva Popovic, James R. Guest, Elizabeth Buccheri, David Idip, Peter J. Mumby

**Affiliations:** 1 Marine Spatial Ecology Lab, School of the Environment, The University of Queensland, St. Lucia, QLD Australia; 2 CSIRO Environment, St. Lucia, QLD Australia; 3 School of Natural and Environmental Sciences, Newcastle University, Newcastle upon Tyne, UK; 4 Palau International Coral Reef Center, Koror, Republic of Palau; 5 Simon F.S. Li Marine Science Laboratory, School of Life Sciences, The Chinese University of Hong Kong, Hong Kong SAR, China; 6 Department of Biosciences, Faculty of Health and Life Sciences, University of Exeter, Exeter, UK; 7 Australian Institute of Marine Science, Townsville, Australia; 8 Palau Automated Land and Resources Information System (PALARIS), Bureau of Budget and Planning, Koror, Republic of Palau

**Keywords:** Population dynamics, Population genetics

## Abstract

As populations decline under climate change and other stressors, survivors become increasingly isolated. Allee effects in such fragmented populations may arise during reproduction, particularly for sessile organisms such as corals, reducing fertilisation potential and increasing the probability of recruitment failure. However, the critical distances between corals needed to maintain viable populations remains uncertain. We experimentally investigated fertilisation patterns in the broadcast spawning coral *Acropora* cf. *hyacinthus* using a manipulated patch, spacing colonies at increasing distances downstream from a central spawning aggregation. Field samples showed a steep decline in fertilisation with increasing intercolonial distance and genetic paternity analysis indicated most parents were located within 3 m of each other. Colony position relative to the current direction also influenced fertilisation success, with 81% of sequenced progeny sired by upstream colonies. Simulations projecting natural population distributions onto a virtual grid, incorporating experimental results, indicated that populations remained well-mixed at typical adult densities. Yet at reduced densities approaching 0.01 colonies m^−2^, reproductive isolation and patchy breeding units emerged. These findings demonstrate that colony isolation substantially impairs reproductive connectivity, with additional influence from the spatial arrangement of colonies. Conservation strategies should prioritise maintaining colony density and spatial connectivity to support viable coral populations on degraded reefs.

## Abstract (web summary)

Experimental and modelling results show that low-density reefs risk reproductive isolation, highlighting the importance of maintaining colony density and spatial connectivity.

## Introduction

Allee effects are a density-dependent phenomenon where reductions in population size and density lead to diminishing individual fitness (e.g., survival, reproduction) and slower population growth rates, risking the possibility of local extinctions^1,2^. In coral reef ecosystems increasingly threatened by climate change, a significant proportion of reefs are now expected to become degraded to a point at which the size and density of populations could become insufficient for successful reproduction^3–5^. Such critical Allee effects are especially pertinent during the external fertilisation process in sessile species that engage in broadcast spawning, where the success of fertilisation is dependent on the quantity and concentration of gametes produced^6,7^. The proximity of conspecific spawning colonies and individual factors like colony size are known to influence gamete output and concentration^8–10^. These high concentrations of gametes are crucial for eggs and sperm to encounter each other for fertilisation during a brief window of a few hours, before wind and currents disperse the gametes^11–13^. When population sizes decline, both the total reproductive output (i.e., the number of eggs, which sets the upper limit for the number of possible embryos) and the proportion of these that are successfully fertilised decrease. If fertilisation failure is severe, it can lead to recruitment failure for the entire reproductive season^14^, adversely affecting the ecosystem's long-term ability to withstand the challenges posed by ocean warming and other stressors^15,16^. Further, recent delineations of cryptic species through genomic analyses suggest that breeding coral populations are at lower densities than earlier estimates^17–19^, which may place some species closer to critical population thresholds than previously thought.

In the dynamic environment of a coral spawning event, where eggs and sperm are suspended in the upper water column, gametic mixing is shaped by a range of abiotic and biotic factors that ultimately influence fertilisation success^20^. In theory, heterogeneity in colony sizes, their spatial arrangement and the directional current of water all contribute to a complex mosaic of conditions that can either facilitate or hinder gamete encounters^11^. Larger individuals, with their greater gamete output, may dominate fertilisation opportunities through increased egg-sperm encounters and gametic mixing. Moreover, the directionality and strength of water currents play a pivotal role in shaping the fate of the spawn slicks and suspensions^11,21^. Currents typically disperse gamete concentrations, decreasing the likelihood of successful fertilisation. However, some physical and hydrodynamic features, such as fronts, eddies, and convergence zones, can contribute to the formation of dense ‘slicks’ of embryos in the hours to days following spawning^22–25^. While these hydrodynamic features can concentrate embryos and decomposing reproductive material, their role in enhancing fertilisation remains unclear, since aggregations may occur too late to significantly influence gamete encounters.

A key opportunity in advancing our understanding of fertilisation patterns lies in the incorporation of parent–offspring relationship data alongside the strategic isolation of targeted colonies from conspecifics. Most studies of fertilisation success in corals and other marine invertebrates rely on statistical associations with biophysical and spatial population parameters, yet they fail to establish direct causal links. For instance, although variations in spatial population parameters (e.g. colony density) may correlate with fertilisation outcomes, without parental lineage data and controlled spatial context, it remains unclear whether these factors actively drive the observed patterns. Additionally, samples taken from spawn slicks to estimate fertilisation success – especially those collected the day after spawning – are prone to biases. This bias arises because unfertilised eggs decompose, resulting in underestimates of initial egg counts and, consequently inflated fertilisation rate estimates^25^. Even when sampling occurs within the fertilisation time window, it can remain challenging to determine whether embryos and their fertilising sperm originated from colonies within the area of interest or nearby reefs^25^. There is also the possibility that embryos are the result of hybridisation between closely related species, complicating intraspecific fertilisation interpretations and raising uncertainty about whether these hybrid embryos will develop successfully beyond the embryo stage^26,27^. Parental assignment methods (e.g. progeny arrays) allow direct characterisation of fertilisation patterns by linking offspring to specific adult colonies. Coupled with spatial data, they offer direct insights into how population characteristics determine reproductive success^28–30^.

Typically, at any given reef, broadcast spawning occurs over one or two nights annually, within a narrow timeframe of 2–3 hours before the cloud of gametes disperses beyond the threshold for effective fertilisation^13,31,32^. Although laboratory-based research has shed much light on species-specific fertilisation mechanisms^26,33–35^, such studies generally ignore the crucial hydrodynamic influences acting on a natural spawn concentration. As such, we know little about how species-specific spawn dynamics relate to spatial population parameters in situ (but see Mumby, et al.^9^, Levitan, et al.^10^, Coma and Lasker^36^, Miller and Mundy^37^, Ricardo, et al.^38^). Understanding thresholds in spatial parameters such as colony spacing and density is not only critical for predicting the reproductive viability of natural reef populations under degradation, but also has direct implications for applied conservation. In particular, restorative approaches such as coral outplanting rely on assumptions about effective fertilisation, yet key knowledge gaps remain regarding how colony spacing and spatial arrangement influence in situ fertilisation rates and, consequently, the long-term contribution of outplanted colonies^39–41^. Gathering empirical data during these annual spawning events presents significant challenges due to their ephemeral nature, nocturnal timing, numerous unknown phenological cues, and the complexity introduced by the simultaneous spawning of multiple species. Here, we aim to address these shortcomings by conducting a manipulative field experiment in which we positioned adult colonies in a radial arrangement at varying distances from a high-density central spawning hub to assess how colony spacing and arrangement influence fertilisation success and potential Allee effects. We utilise paternity assignments based on next-generation sequencing of adult colonies and individual larval progeny from the generated spawn plume to understand how gametic mixing, colony distance, directionality, colony size and genetic relatedness influence fertilisation outcomes.

## Methods

### Coral collection and experimental site selection

Here we assessed fertilisation success of the reef-building table coral *Acropora* cf. *hyacinthus* in Palau, Micronesia. *Acropora* cf. *hyacinthus* is a large hermaphroditic simultaneous broadcast spawning coral found in a range of physical environments and its fast growth allows it to facilitate reef recovery^42^. We note that *A. hyacinthus* is referred to as *A*. cf. *hyacinthus* throughout the text, as its taxonomic status was under revision at the time of the study. This species has since been reassigned to *A*. *tersa*^43^, and morphologically matches the lineage reported as *A. hyacinthus C* in Ladner and Palumbi^44^. Most corals, including *A*. cf. *hyacinthus*, spawned on the inshore reefs adjacent to our reef site on the 1 April 2023, five days before the full moon and earlier than anticipated, coinciding with warm doldrums (~33 °C water temperature). On the 2 April 2023, approximately 95% of colonies of *A*. cf. *hyacinthus* (Dana, 1846) were gravid at the offshore Uchelbeluu Reef (7.2591° N, 134.5295° E), and 25 medium-sized genetically distinct colonies were subsequently collected, allowing at least 5 m distance between colonies to prevent these being from the same genet^45^. Individuals were returned to their natal reef following the experiment.

**Fig. 1:**
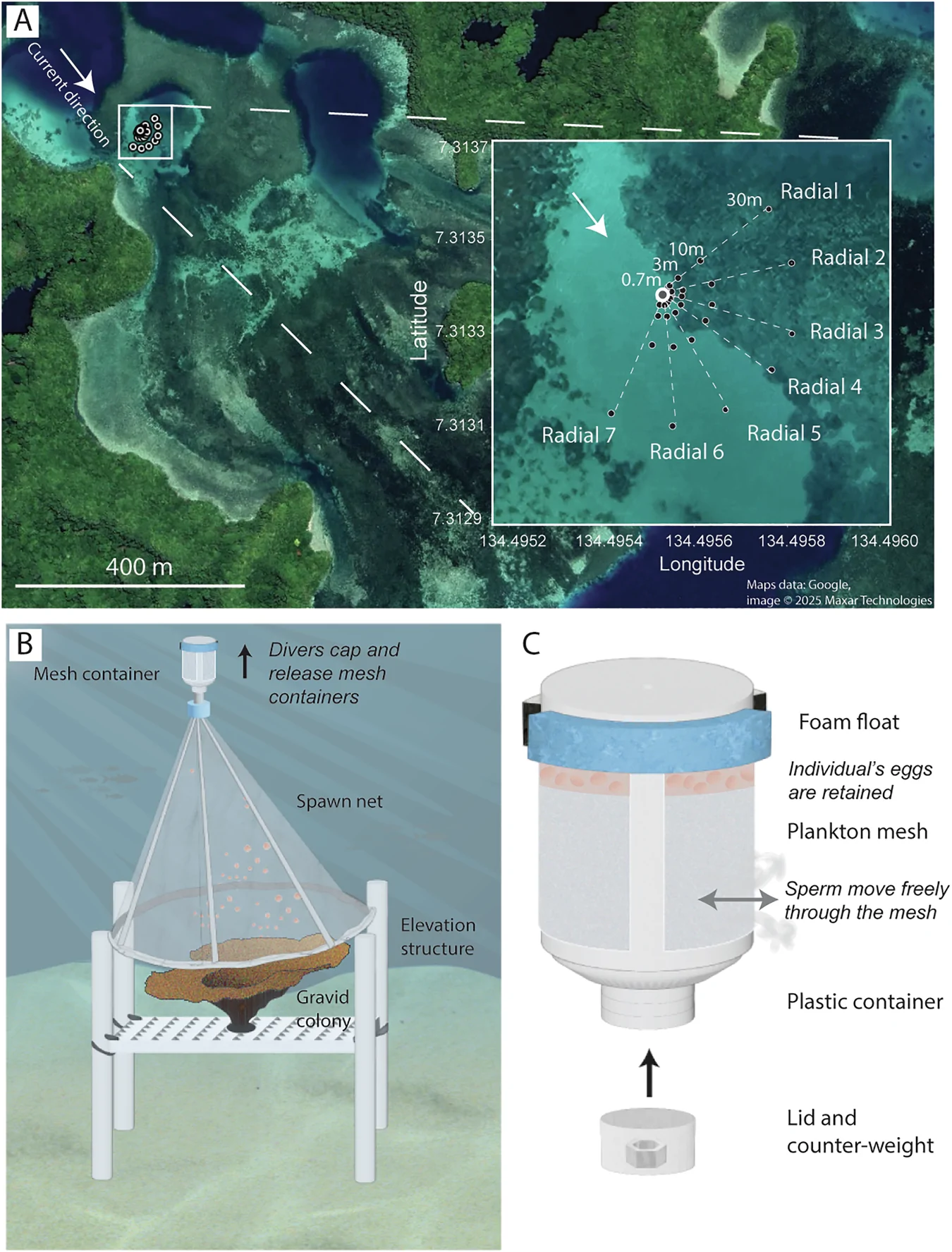
Experimental design and coral spawn catcher apparatus designs. **A** Map of Ngermid Bay showing the location of the study site. The inset illustrates the clustered radial arrangement of adult gravid colonies of *Acropora* cf. *hyacinthus* within the bay, with colonies positioned along the radial lines (denoted by black points) at distances of 0.7, 3, 10, 30 m from the central patch (large grey point). The arrows denote the current direction. Map data from Google, image © 2025 Maxar Technologies. **B** Design diagrams of spawning catcher apparatus used to funnel the egg-sperm bundles to the mesh containers. Divers then release mesh containers which float within the gamete plume at the water’s surface produced from all colonies within the experimental patch. Coral image adapted from Integration and Application Network (ian.umces.edu/media-library), licensed under CC BY-SA 4.0. **C** The design of the mesh containers. The individual colony’s eggs are retained within the container whereas the sperm moves freely through the mesh. Illustration modified from Mumby et al.^9^.

To ensure a controlled experiment, a field site was selected in a sandy area at the entrance to Ngermid Bay (also known as Nikko Bay, 7.3135° N, 134.4960° E) (Fig. 1a), a complex of channels and bays on the southeastern side of Koror, Palau, known for its diverse coral communities^46^. Ngermid Bay was selected because *Acropora* cf. *hyacinthus*, our target species, was absent from the area. Although Ngermid Bay hosts other corals such as *Porites* (56%) and *Goniopora* (9%), *Acropora* are rare (~1%)^47^. Moreover, as nearby inshore colonies of *A*. cf. *hyacinthus* spawned earlier and were not observed within the bay, there was a reduced risk of gamete contamination. Outgoing tidal currents likely advected gametes from natural *Acropora* populations observed at the mouth of the bay away from our experimental gamete cloud^47^, further minimising hybridisation risk.

### Experimental design

The experiment was designed to assess the effect of intercolonial spacing and spatial arrangement of spawning colonies on fertilisation success, utilising several radial lines extending from a central high-density patch of gravid corals in a semi-circle shape (Fig. 1A). The experimental arrangement was strategically oriented such that the central radial extensions were positioned downstream relative to a high-density aggregation of colonies, aligning with the anticipated current direction characteristic of the bay. Seventeen *A*. cf. *hyacinthus* colonies (of 16 unique genotypes) were selected haphazardly and arranged in the central patch, each spaced 0.7 m apart measured from the centre of each colony, broadly corresponding to intercolonial distances found for adults of this species in nearby high-population density reefs^38^. Extending from this semi-circle, seven radial lines were marked, with reference points for coral placement at 0.7, 3, 10, and 30 m intervals along axes set at ~26° increments (Fig. 1A). These radial lines corresponded to the true bearings of 51°, 77°, 101°, 124°, 148°, 173°, 201°, with the measured current direction during the experiment at 143°, falling between radial line 4 and 5 (described below). To avoid potential smothering of sand, coral colonies were elevated approximately 30 cm above the seabed using structures composed of 0.7 m pipes and egg crate material, all of which were georeferenced using handheld GNSS receivers (Fig. 1A). To mitigate the risk of cross-fertilisation between adjacent colonies within and between radial lines, larger colonies were fragmented and strategically distributed where possible across nearby positions, typically along that colony’s corresponding radial line. This arrangement aimed to minimise the potential for cross-fertilisation among nearby positions so maximum parental distances could be assessed. The average diameter of the coral colonies used in the experiment was 32.6 ± 12.9 cm, slightly larger than the 26.0 ± 13.1 cm diameter mature colonies observed in natural populations on a nearby offshore reef^38^.

Immediately prior to coral spawning, coral spawn catchers were positioned approximately 20 cm above the colonies along the radial lines 2 to 7 (Fig. 1B), as well as above four randomly selected central colonies. The spawn catcher nets (~100 µm mesh size) channelled the egg-sperm bundles into detachable 270 mL containers. Approximately 46% of each container's wall was removed and replaced with a 250 µm mesh, allowing sperm to diffuse freely while retaining the eggs of individual colonies (herein referred to as ‘mesh containers’). As a result, eggs from tracked colonies (hereafter referred to as egg donors) were fertilised by sperm from initially unidentified colonies (hereafter referred to as fertilising sperm donors). Although all in situ fertilisation collection methods introduce some degree of artefact (see review by Levitan^48^), egg containment is often necessary in studies of small populations where natural egg retrieval via plankton sampling is infeasible or where mixing with eggs from other species is a concern. Previous work has shown that sperm can move freely through the plankton mesh of the containers, with the resulting fertilisation success within the range of mesh-free controls^9,38^. To facilitate tracking, glowsticks were affixed to the mesh containers using cable ties. Each container was modified with flotation on the top and a bolt counterweight in the base, ensuring the containers floated within the gamete plume at the water’s surface but also remained vertically oriented, with ~10 cm submerged in the water column (Fig. 1C).

During the predicted coral spawning window, divers entered the water at ~18:00 and monitored corals along the patch using red beam dive torches. All but one monitored colony commenced spawning within a 12 min period of each other. Once individual colonies were spawning, a single mesh container was removed from the spawn catcher, capped, the glowsticks activated, and mesh containers released. Releasing containers as each colony began spawning allowed temporal variation in spawning across the patch to be captured. Due to logistical constraints, retrieval was conducted via kayak in shallow areas inaccessible by boat approximately 80–90 min after release, with 25 of 28 containers successfully retrieved. Following collection, the mesh containers remained submerged in the ambient water post-collection until they were processed in the laboratory approximately 30 min later. Although this collection method might have prolonged gamete contact time, any additional fertilisation resulting from extended exposure is assumed to be negligible given rapid sperm dilution that had already occurred before collection^31^.

On the night of the experiment, the tide was at mid-ebb (0.67 amplitude), flowing SSE toward the bay entrance. The site was largely sheltered from the rock islands from the prevailing 2.4-knot NNW offshore wind, resulting in the gamete concentrations and mesh containers being predominantly driven by tidal currents. Water depth downstream of the experimental setup varied between 1 and 3 m, with mesh containers drifting at a mean velocity of >0.21 m s^−1^ (*n* = 3), placing these velocities in the 97th percentile of water speeds recorded by the Marotte tilt-meter during a two-day deployment under a comparable tidal regime. Additionally, tracer dye (fluorescein) releases were conducted at the centre point of the site during comparable tidal cycles as the main experiment to understand the movement and dispersion properties likely acting on the gamete plume. Two grams of the dye was mixed into 2 L of seawater and released from the benthos by a diver. The plume was tracked using an Unmanned Autonomous Vehicle (UAV: DJI Mini 3 Pro) with an interval rate of 2 s. For image processing, the UAV images were converted to JPEG files, and the length of the research vessel was used as a reference to set the image scale. The images were split by colour channels using the package cv2 in Python (v. 3.8.8), and an index was used to maximise the signal of the dye relative to background features, based on the spectrophotometric profile described in Ricardo, et al.^38^

$$\frac{{Green}-{Blue}}{{Green}+{Blue}}$$

where *Green* and *Blue* represent the green and blue colour channels, respectively.

Current direction was determined from the release location of each container and the recorded waypoints during retrieval, indicating an SSE current direction (143°) (Fig. S1). Container trajectories deviated by approximately ±1.2° circular SD from the mean current direction, and observations by kayakers tracking the containers confirmed minimal variation in their paths throughout the release period.

### Bulk fertilisation success

Samples for bulk fertilisation success counts were fixed with 4% buffered formalin in filtered seawater containing 10 g L^−1^ sodium β-glycerophosphate at a ratio of 1:4 fixative to sample volume, approximately ~150 min after spawning when the developing embryos were observed to reach the four-cell cleavage stage. These samples were later assessed under a dissection microscope by five assessors for fertilisation success, recorded as the number of cleaved embryos as a proportion of total initial eggs. Between 85 to 500 eggs or embryos were counted per sample depending on the level of fertilisation success observed in the sample.

A number of controls were conducted on a nearby vessel using bundles collected shortly after the release of the mesh containers, by attaching closed containers to spawn catchers of two randomly selected colonies. First, two separate one-to-one crosses were conducted, which resulted in 71–80% fertilisation success, indicating substantial gamete compatibility. Next, two self-fertilisation controls were conducted with one sample resulting in unrealistically high self-fertilisation (18.1%) in contrast to the other sample (1.0%). Together with the results of the paternity assignments (described below), and previous self-fertilisation reported of this species at this location (0.1%)^38^, it is suggested that this container was likely contaminated during the collection processes while the container was uncapped and attached to the spawn catcher.

### Genetic sampling and sequencing

A small amount of tissue from each of the adult colonies was collected into individual ziplock bags. The tissue for each individual was further divided into two and placed in 1 mL of 100% ethanol in a 2 mL Eppendorf and placed in a −80 °C freezer. To assess paternity, genetic tests were conducted on individual larvae allowing determination of the fertilising sperm donors from the sample from known egg donor colonies following Ricardo, et al.^28^. Fertilised eggs from the mesh containers were separated individually and allowed to develop until 36–42 h old and then pipetted with 10 µL seawater in 100 µL of 100% Ethanol. After 24 hr, a 50% ethanol exchange was conducted on each sample to further remove seawater and contaminants. Samples were shipped back to the University of Queensland, Australia (CITES permit number PW23-079).

A total of 82 adult colony (*n* = 2 per colony) and 139 larval samples were transferred to 96-well plates (Eppendorf, TwinTec®, skirted, 150 μL, PCR clean), capped (Eppendorf, Cap Strips, 8-strips, domed), and sent to Diversity Arrays Technology (DArTSeq; Canberra, Australia) for DNA extraction, library preparation, sequencing and single nucleotide polymorphisms (SNP) identification. DArTSeq sequencing is a technique that employs pairs of restriction enzymes to digest genomic DNA, similar to other site-associated reduced representation methods (e.g., RADseq) enabling the identification of genome-wide SNP markers. Sequencing was carried out on an Illumina short-read platform to produce raw “sequence tags” of approximately 75 base pairs. Samples were aligned to two reference genomes for *Acropora hyacinthus*: *Acropora_hyacinthus_*chrsv1 (accession number: GCA_020536085.1; Palumbi^49^) and *Acropora_hyacinthus_v002* (available at http://ahya.reefgenomics.org/; ReFuGe^50^). The raw sequence reads were processed following DArT’s proprietary variant calling pipeline. Raw reads were filtered based on sequence quality, especially in the barcode region, truncated to 69 base pairs, and grouped by sequence similarity. Subsequently, a series of filters was applied to select sequence tags that contain reliable SNP markers. One-third of the samples were processed twice as technical replicates, starting from DNA and using independent adaptors, through to allelic calls. Then, a series of proprietary filters were applied to select the sequence tags that contain reliable variants. High-quality markers with low error rates were primarily selected using scoring consistency (repeatability), assessed using technical replicates.

Raw SNP sequencing data underwent standard filtering processes for paternity assignments following Premachandra, et al.^51^, and Jenkins, et al.^52^. Initially 50,405 SNPs were identified, and after removing monomorphic loci, 50,236 SNPs remained. Next, an initial filtering step was applied to remove loci > 50% missing data and to exclude loci with a minor allele frequency (MAF) < 0.05 to minimise the possibility of scoring sequencing errors as rare minor alleles. Loci were then further filtered based on read depth at a minimum mean threshold of >3 reads. A stricter filtering step was then applied to retain only loci with a call rate >95% and a mean read depth of >20, followed by removal of loci with excessively high read depth based on a three-standard-deviation range. Secondary loci were also eliminated using a random selection method, and loci were assessed for reproducibility, with those <95% threshold discarded. Finally, loci deviating significantly from Hardy-Weinberg equilibrium (HWE) were filtered and false discovery rate (FDR) multiple testing corrections applied. The final dataset consisted of 1,328 SNPs across 81 adult samples and 120 larval samples, which was used in downstream analyses. Prior to paternity assignments, final filtering was conducted following Premachandra, et al.^51^ to remove mean MAF values < 0.2. Typically, only low numbers of high-quality SNPs are required for accurate parentage assignments^51,53^.

#### Paternity assignments

Paternity assignments were conducted on *A*. cf. *hyacinthus* larval samples with known maternal genotypes at the time of egg capture. Paternity assignments were carried out using the software CERVUS 3.0.7^54^. CERVUS is a simulation-based approach that uses likelihood ratios to assign parentage. The simulation-based approach is relatively robust to null alleles and genotyping errors. Simulations were based on allele frequencies of the experiment population; 20,000 offspring, a 5% error rate, and 95% sampled candidate fathers were used as inputs. A 95% ‘sampled candidate fathers’ rate was chosen to account for a few individuals that were not successfully sequenced. Since CERVUS cannot identify unsampled candidate parents during assignments, this uncertainty is reflected in the confidence levels of the assignments. The assignments were conducted under the ‘strict’ (95%) confidence levels using log-likelihood ratio (LOD) scores. Assignments for larvae were removed when the sperm donor could result from multiple positions i.e. from fragments along the radial lines.

#### Population structure

Clones within the sample set, including fragments used within the radial lines, were assessed using COLONY v2.0.7.1^55^, a maximum likelihood-based software designed for inferring sibship, parentage, and clonemates from multilocus genotypic data. The analysis was run on 81 multilocus genotypes using the full-likelihood (FL) method, with medium run length and high precision settings. The dataset was specified as monoecious, diploid, with clonal inference enabled. Clone assignment in COLONY is achieved via a two-step process: first, full-sib families are inferred, and then clone groups are delineated within these families by maximising the likelihood of shared multilocus genotypes, accounting for potential mistyping. All clone groups were identified with high confidence (most with posterior probabilities of 1.000). Clones and replicates were removed before conducting the population structure analysis, ensuring that only unique genotypes were compared.

To assess the population genetic structure within the population, we performed Principal Component Analysis (PCA) on quality-filtered allele frequency data using the package *ade4*^56^, in R (v 4.4.1). PCA was conducted with centring and scaling, and the first 18 principal components were analysed which represented ~90% of the variance. To test for population clustering, we applied k-means clustering, and the number of clusters was assessed using the Elbow Method (total within-cluster sum of squares) and Silhouette scores. Additionally, we performed STRUCTURE analysis within the package *dartR*, using an admixture model with correlated allele frequencies. STRUCTURE was run with a burn-in of 10,000 iterations, followed by 50,000 MCMC replications, testing K = 1 to K = 5, with two replicates per K. The optimal K was determined using the Evanno method^57^. To further assess genetic differentiation, we conducted a PERMANOVA in the package *vegan*^58^, testing whether genetic distances differed significantly between groups, with 9,999 permutations to evaluate statistical significance.

### Statistics and reproducibility

Where necessary, fertilisation success data were subsampled to balance differences in sample sizes between mesh containers, with the containers treated as the experimental unit. The relationship between fertilisation success with degree deviation (i.e., 0°) from directly downstream of the central patch (*angle*, continuous, modelled non-linearly) and distance from the central patch (*dist_center*, continuous) was analysed using a Generalized Additive Mixed Model (GAMM) with a binomial family:

$${{{\rm{logit}}}}\left({{{{\rm{\pi }}}}}_{{ij}}\right)={{{{\rm{\beta }}}}}_{0}+{{{{\rm{f}}}}}_{1}({{angle}}_{i})+{{{{\rm{\beta }}}}}_{1}\cdot {{dist}{{{\rm{\_}}}}{center}}_{i}+{u}_{j}$$

where π_*ij*_ represents the predicted probability of fertilisation success for an individual observation *i* within a specific mesh container *j*. The term *u*_*j*_ represents an observation-level random intercept to account for overdispersion^59^. All analyses were two-sided. The GAMM was implemented in R (v. 4.4.1) using the package *mgcv*^60^. The appropriate dimension of the smoother term *k* was tested using residual-based diagnostics, and model diagnostics are shown in Fig. S1. Additionally, three colonies located along the radial lines that shared a clonemate with an individual in the high-density patch were adjusted in the model to account for their relatively reduced exposure to unique sperm sources. However, including this adjustment had negligible effect on model performance and reduced parsimony, so it was not retained in the final model.

For paternity assignments, observed pairwise crosses were first analysed separately for the factors of parental distances and deviation from the current direction. Distances between assigned parents were calculated with and without weighting using:

$${w}_{i}=\frac{1}{{N}_{{\mbox{larvae}}}}$$

where *N*_larvae_ was the number of larvae per container. Weighting was applied to prevent an overrepresentation of highly sequenced mesh containers, though this approach can disproportionately amplify trends in mesh containers with low sample sizes. A Kolmogorov-Smirnov (K-S) test was used to assess if the pairwise-cross distance distribution differed from random chance based on the distance distribution between candidate adult colonies. Additionally, a Rayleigh’s test for uniformity was conducted to assess whether the spatial distribution of colonies relative to the current direction deviated from uniformity.

Next, the statistical relationships between individual or population parameters and assigned parent colonies were examined. Observed pairwise combinations of parents were treated as count data and compared against every possible pairwise combination of candidate adult colonies in the experiment, excluding unlikely combinations such as those between known genotype fragments. Given the combination of true and false zeros – where total embryos in a container after fertilisation exceeded those larvae eventually sequenced – in the dataset^61^, relationships with predictor variables were assessed using zero-inflated negative binomial model using the package *glmmTMB*^62^. To account for excess zeros, including both true and false zeros, the zero-inflation component was tested using three approaches: (1) total embryos per container as a predictor, (2) a constant (intercept-only model), and (3) without the zero-inflation component altogether. Treatments included distance between parent colonies (*distance*), angle of parent colonies relative to upstream/downstream direction (*angle*), colony size (*col_size*) and genetic relatedness (*gen_dist*). We hypothesised: (1) a negative relationship between colony distance and fertilisation success, moderated by colony angle, (2) a positive effect of colony size, and (3) a non-linear (quadratic) effect of genetic distance, peaking at intermediate values. To address collinearity, covariates were centred and scaled, resulting in a Variance Inflation Factor (VIF) < 2 for all predictors.

A global model was constructed with biologically meaningful interactions and factors, incorporating an offset term for total fertilisation (*total_fert*) in each mesh container. The offset helped mitigate biases introduced by unequal sequencing effort across mesh containers. The model was specified as follows:

$${{\mathrm{ln}}}\left({\mu }_{i}\right)= 	\, {\beta }_{0}+{\beta }_{1}\cdot {distanc}{e}_{i}\times {\beta }_{2}\cdot {angl}{e}_{i}+{\beta }_{3}\cdot {poly}\left({{gen}{{{\rm{\_}}}}{dist}}_{i},1\right) \\ 	 +{\beta }_{4}\cdot {poly}\left({{gen}{{{\rm{\_}}}}{dist}}_{i},2\right)+{\beta }_{5}\cdot {{col}{{{\rm{\_}}}}{size}}_{i}+{{\mathrm{ln}}}\left({{total}{{{\rm{\_}}}}{fert}}_{i}\right)$$

and the zero-inflation component as:

$${logit}\left({\pi }_{i}\right)={\gamma }_{0}+{\gamma }_{1}\cdot {{total}{{{\rm{\_}}}}{fert}}_{i}$$

where *μ*_*i*_ represents the expected number of fertilised embryos for a given observation *i*. To identify the most appropriate error distribution and assess the need for a zero-inflation component, the global model was first fitted and compared across Poisson and negative binomial distributions, with and without zero-inflation, using AIC. Based on these comparisons, the most parsimonious model was a negative binomial distribution without a zero-inflation term. Model selection was then carried out using the *dredge* function from the MuMIn package^63^, and a set of candidate models with ΔAIC < 2 were retained for model averaging. Across these, distance and angle consistently appeared as additive predictors, while the interaction and other covariates had low inclusion frequencies and weak support. Model assumptions were evaluated using the DHARMa package^64^, and there was no clear indication of model assumption violations.

### Gamete plume mixing simulations

To explore the potential for gametic mixing during fertilisation within realistic population spatial structures, we simulated a 100 × 100 m virtual grid at 0.1 m resolution. Each grid was populated 200 times at varying population densities for each of three spatial distribution types using the R package *spatstat*. First, a random (Poisson) process was implemented. Next, we used two spatial distributions derived from natural reef populations near the study site described in Ricardo, et al.^38^. Belt transect data from reef crest (*n* = 3) and reef slope (*n* = 1) habitats for adult (>10 cm) *A*. cf. *hyacinthus* were fitted to Poisson, Thomas, and Matérn cluster models. Across reef crest transects, Matérn and Thomas cluster models produced very similar AIC values, with Matérn being marginally better supported in some cases (ΔAIC ≤ 1). Both Thomas and Matérn cluster processes provided a good fit to the reef slope data, but the Matérn model was marginally better supported (ΔAIC = 1.6) and consequently used for all habitats. The best-fit model parameters were extracted and used to simulate colony distributions for ‘reef crest’ and ‘reef slope’ simulations. Euclidean distances and angles for all pairwise combinations of adult colonies within the grid were computed. The expected number of fertilised embryos per pair was determined using the coefficients for distance and angle from the statistical model of our field experiments, scaled by the total expected number of embryos per egg donor (under the assumption that each colony releases approximately 10⁵ eggs). During each simulation, this prediction was used to derive the probability of fertilisation occurring at least once between adult pairs, which was implemented as the success probability of a Bernoulli trial: a pair was recorded as successful with probability P( ≥ 1 event) and unsuccessful otherwise. Simulations were run across a gradient of adult densities, ranging from low-density (highly degraded reefs, 0.0003 colonies m^−2^; Zayasu and Suzuki^65^) to densities observed on healthy reefs (0.3 colonies m^−2^). Although species-specific density benchmarks are rarely reported, 0.3 colonies m^−2^ reflects Palauan sites that have largely returned to pre-disturbance colony densities and are considered to exhibit high coral cover within the study region^66^. ‘Partners per individual’ and the proportion of ‘isolated individuals’ were extracted from each run as metrics to indicate gametic mixing. The increase in colony density required for the predicted mean ‘partners per individual’ to double was derived, calculated as *ln*(2)/*b*, where *b* is the exponential growth rate.

### Reporting summary

Further information on research design is available in the Nature Portfolio Reporting Summary linked to this article.

## Results

### Bulk fertilisation success

Mean fertilisation success across all mesh containers (*n* = 25) was 32.8%, ranging from 0 to 95%. Fertilisation success (mean ± SD) within the central patch was 50.6 ± 34.0%, whereas fertilisation across the radial lines averaged 29.0% and ranged from 0 to 95%. The samples with the highest fertilisation success, exceeding 90%, were all located directly downstream of the central patch.

**Fig. 2:**
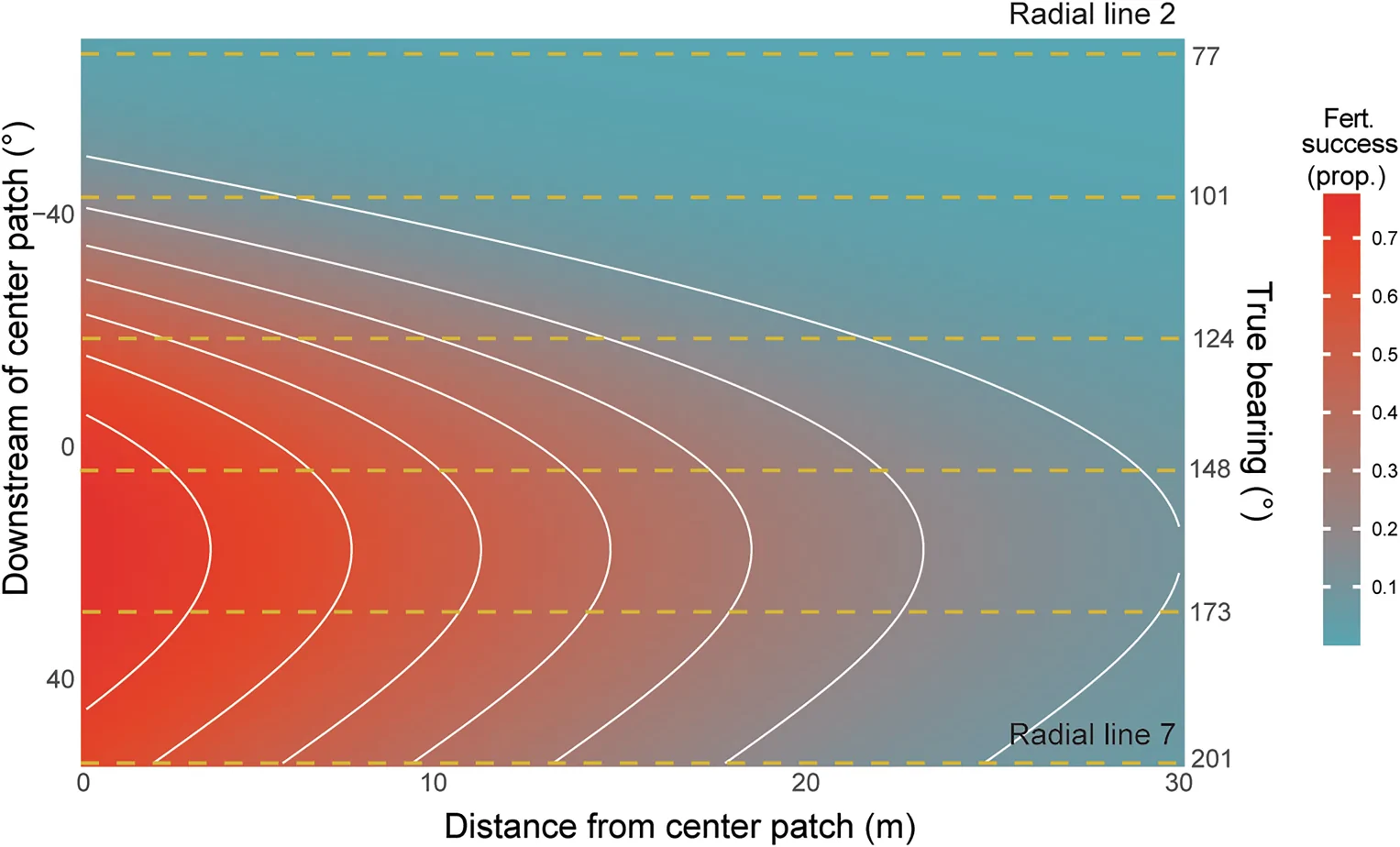
Effect of colony distance and orientation relative to the current on fertilisation success. The effect of distance of colonies (*n* = 21) along each radial line relative to the central spawning patch, and their spatial position relative to the current advection line (0°) on fertilisation success (proportion fertilised), derived from the bulk fertilisation success analysis. White lines represent every 0.1 fertilisation success proportion interval. Orange lines denote the radiating lines in the experimental patch. Only radial lines 2 to 7 were assessed for fertilisation success.

Model results from the GAMM analysis revealed there were significant effects of colony’s distance from the central patch and its angle relative to the downstream current direction on fertilisation success. Specifically, distance from the centre of the patch resulted in a negative association with fertilisation success (β = −0.1168, *p* = 0.002), corresponding to an ~11% decrease in the odds of fertilisation for every additional metre away from the centre, while holding ‘angle’ constant. Furthermore, the angle of the colony from downstream current direction was significant (F = 15.33, *p* < 0.001), indicating that fertilisation success peaked between the radial lines 5 and 6 following the main current direction (Fig. 2). Model residuals showed high variability between samples (adjusted R^2^ = 0.60), including those in close proximity, suggesting spatial patchiness. Some of this variability is likely attributed to cross-fertilisation between colonies positioned on radial lines (see paternity assignments results below).

### Paternity assignments

**Fig. 3:**
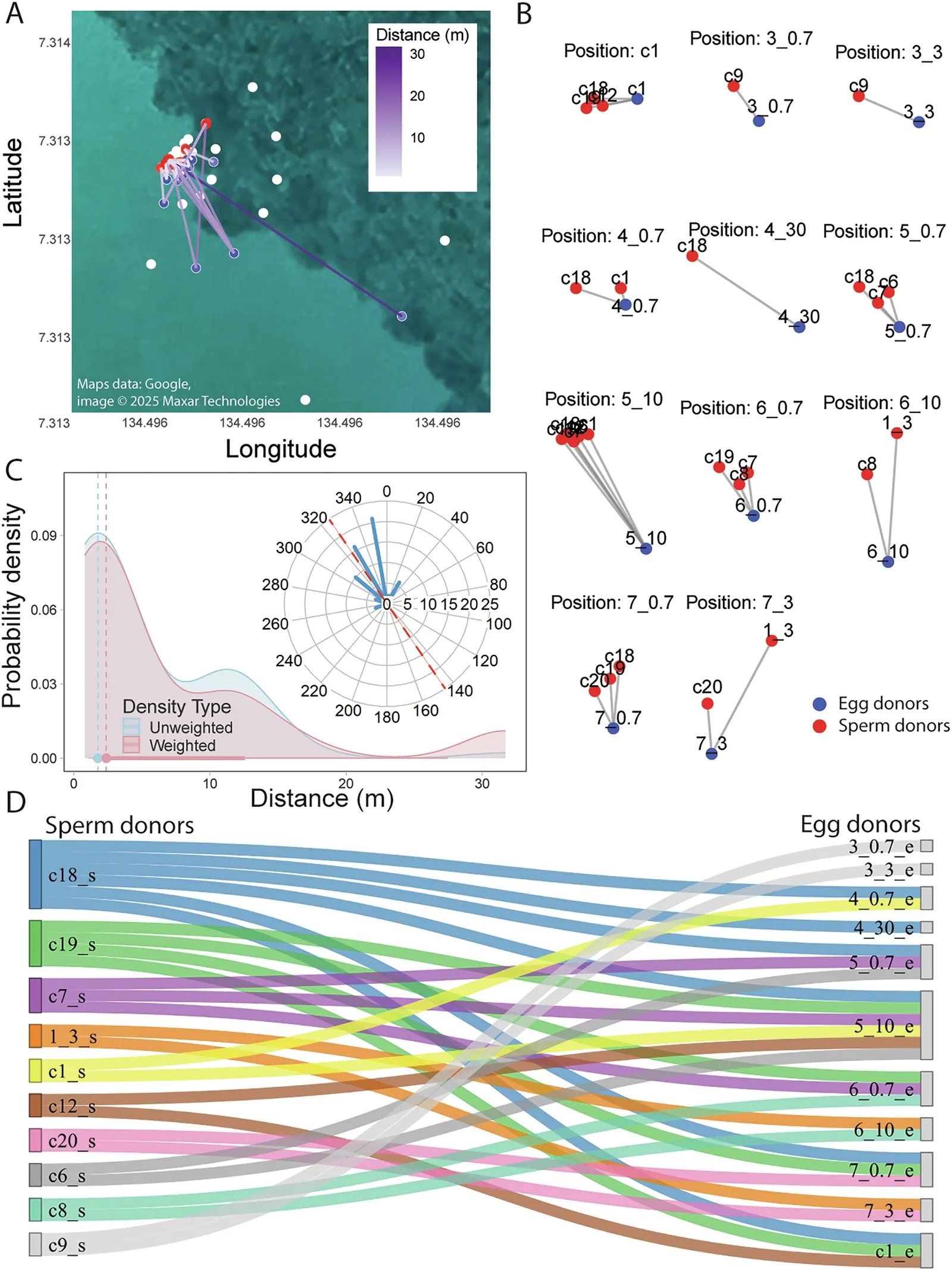
Pairwise analysis from paternity assignments between egg donors (*n* = 11) and fertilising sperm donors (*n* = 10). **A** All pairwise crosses within the experimental patch. Egg donors = blue nodes. Fertilising sperm donors = red nodes. A small amount of jitter was added to the sperm donors for visibility. Map data from Google, image © 2025 Maxar Technologies. **B** Spatial arrangement of all breeding units within the patch relative to the egg donor positions (blue). Red denotes sperm donor positions. Peripheral colonies are labelled in the format ‘RadialLine_Distance_Donor’ where is the distance from central patch, and for central patch colonies ‘cID_Donor’, where “c” indicates a central patch colony. **C** Weighted and unweighted intercolonial distances between pairwise crosses. Inset: Direction of fertilising sperm donors relative to egg donors. True north = 0. Current direction signified by the red line. **D** Sankey diagram illustrating unique pairwise crosses between identified sperm donors and known egg donor colonies. Colony labels correspond to each individual’s spatial position within the experimental design, as described above.

After excluding larvae with ambiguous paternity, a total of 62 sequenced larvae were assigned to a unique sperm donor genotype with high confidence (>95%). The median distance between parental crosses was 1.8 m, whereas the median weighted distance was 2.4 m (Fig. 3a, c). When the pairwise cross distributions were compared against the distributions of the distances between individual adult colonies, there was a significant difference (unweighted: D = 0.459, *p* < 0.001; weighted: D = 0.964, *p* < 0.001), with a preference toward lower crossing distances than expected by chance. Bearings of fertilising sperm donors relative to their respective egg donor were predominantly from 300 to 20 degrees (NW-N), aligning closely with upstream current direction (323 degrees) (Fig. 3c). A Rayleigh test of uniformity, which does not account for other covariates, indicated strong directionality of the pairwise crosses (R = 0.866, *p* < 0.001), with 81% of sequenced progeny sired by colonies located within 90° of the upstream direction. This directionality was supported by tracer dye releases which revealed the gametes may form longer plumes in the direction of the current under higher current velocities (Fig. S2).

Not all colonies participated in cross-fertilisation; even gametes from some colonies in very close proximity sometimes failed to cross-fertilise. Among the participants, 10 colonies contributed sperm that successfully fertilised eggs from 11 colonies (Fig. 3B, D). There were 28 unique pairwise crosses, with each egg-donor colony fertilised by an average of 2.8 sperm-donor colonies. However, reproductive success was unevenly distributed such that the top three sperm donors fertilised 67% of participating egg providers at least once (Fig. 3D).

The model-averaged statistical model describing the number of embryos observed per pairwise cross between parent colonies included all predictors (effect plots shown in Fig. S3); with the only statistically significant predictors being distance and colony positions relative to current direction. The number of embryos per pairwise crosses decreased as the distance between colonies increased (β = −0.130, *p* = 0.004), while colonies positioned more favourably relative to current resulted in a greater number of embryos per pairwise crosses (β = 3.005, *p* = 0.029). Based on parental genetic relatedness, self-fertilisation was identified in five larvae, each from a different egg donor, representing 4.6% of the population. Paternity assignment analysis also revealed variability in the bulk fertilisation analysis was largely driven by cross-fertilisation between radial lines. There was limited evidence of population structure among the collected colonies within the small spatial scale of the collection site, validating that sampled colonies belong to a single taxon (Note S1 and Fig. S4).

### Gamete plume mixing simulations

**Fig. 4:**
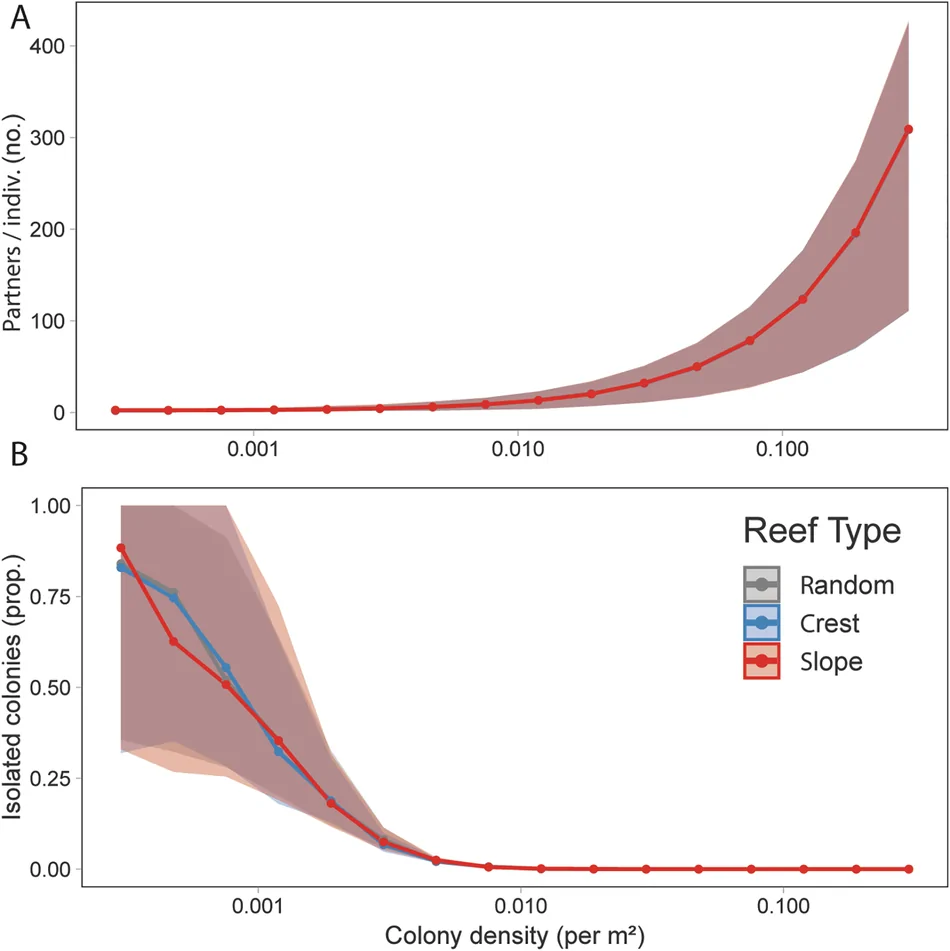
Simulated relationships between colony density and key gametic mixing metrics across different reef types in an area of 1 hectare (10,000 m^2^). **A** Number of reproductive partners per individual, and **B** proportion of isolated colonies relative to the total number of colonies on the grid, as a function of colony density (per m^2^). Shaded areas represent the 95% percentile range. Data are shown for three spatial dispersion patterns: random (grey), reef crest (blue), and reef slope (red).

All three spatial dispersion patterns (random, reef crest and reef slope) exhibited similar trends with gametic mixing metrics and were subsequently pooled (Fig. 4, S5). The number of parental partners per individual followed an exponential increase with adult density (Fig. 4a), with the doubling density of the curve at 0.084 colonies m^−2^, with few partners per group occurring at densities under ~0.01 colonies m^−2^. Surprisingly, few colonies were completely isolated from cross-fertilisation until densities were very low at ~ 0.01 colonies m^−2^ (Fig. 4b).

## Discussion

By combining two complementary fertilisation assessment methods with a spatial manipulation experiment designed to evaluate reproductive success, we reveal strong spatial constraints on coral fertilisation. We find that fertilisation in broadcast spawning corals is related to the spatial positioning of the adult colonies, particularly their alignment relative to prevailing tidal currents and their proximity to high-density spawning patches, with the highest fertilisation occurring parallel to the current direction.

Paternity analyses provide critical insights into gamete interactions within the gamete cloud, capturing distances between mating pairs not readily apparent in bulk fertilisation estimates^67^. The median fertilisation distance between assigned parent pairs was 3–4 m. Although the median fertilisation distance is influenced by our experimental design (which included a higher density of closely spaced colonies), it aligns with predictions from biophysical models and previous empirical studies, and indicates that broadcast spawning corals isolated by beyond 20 m are limited in their potential for fertilisation^9,10,12,38^. Surprisingly, this maximum distance of fertilisation limitation in spawning corals is similar to brooding corals that utilise internal fertilisation, despite spawning species' gametes often dispersing hundreds of metres during the fertilisation window. Using paternity assignments, Warner, et al.^30^ found a maximum distance of 17 m for the brooding coral *Seriatopora*, with most fertilisation occurring within 10 m. Likewise in brooding octocorals, paternity analyses found the maximum distance of 12 m despite the relationship with proximity being less clear^68^. Our findings underscore the risk that depleted populations, with increased distances between colonies, are highly susceptible to component Allee effects that significantly impair fertilisation during spawning events.

The spatial orientation of spawning coral colonies relative to tidal currents and the presence of upstream conspecifics substantially influences fertilisation success. Under the observed conditions, advection and longitudinal spreading predominated, while transverse dispersion was minimal (hydrodynamic conditions illustrated in Fig. S2), colonies positioned downstream of one another within a patch experienced greater fertilisation success. Paternity analyses revealed that egg donor colonies were predominantly fertilised by upstream sperm donor colonies, a result consistent with previous findings^11^, and attributed to current-mediated sperm transport dynamics. The high-density upstream patch of spawning corals served as the primary source of sperm for downstream egg donor colonies, supporting the anisotropic nature of fertilisation. On natural reef slopes, this alignment with current direction may arise due to topographical constraints that impose linear colony arrangements along shore-parallel tidal currents^23^. However, in environments where currents shift onshore into lagoons or offshore into deeper waters under changing wind conditions, the directional advantage can be diminished and alter fertilisation success patterns^9^. Thus, the spatial arrangement of colonies relative to prevailing currents can create major fertilisation asymmetries, underscoring the importance of considering hydrodynamics in reproductive connectivity on coral reefs.

Interestingly, although the most parsimonious model of the paternity assignments included genetic relatedness and colony size, we did not find strong evidence supporting their effect on fertilisation success. Intermediate genetic distances appeared to align with higher fertilisation success in some cases, yet this association was not statistically significant. Likewise, and contrary to expectations, individual colony size also showed no statistically significant effect. Colony size is typically proportionate to gamete production and larger colonies were expected to dominate fertilisation outcomes. Previous modelling exercises have highlighted mean population colony size as a major determinant of fertilisation success in broadcast-spawning corals, given established size-fecundity relationships^8,12,38,69^. However, while population-level mean colony size may influence fertilisation, individual colony size showed minimal impact within our experimental design. Instead, we observed that colony distance and relative positioning concerning current direction were more influential factors. Other individual gamete characteristics such as viability and competition were not assessed and considered outside of the scope of the study.

Limited gamete mixing and connectivity among nearby individuals at lower population densities restricts the potential for successful reproductive partnerships and thereby may act to constrain genetic diversity. Gametic mixing patterns across various colony densities were evaluated using simulations parameterised with empirically derived pairwise distances between parents from the experiment, under spatial distributions typical of *A*. cf. *hyacinthus* reef environments, incorporating both observed natural patterns and random distributions. These simulations indicated that as colony density increased, the number of partners per individual (breeding units) increased markedly. This increased acceleration in mixing was observed above 0.01 colonies m^−2^, suggesting conditions that may facilitate broader gamete exchange and connectivity, potentially resembling a well-mixed gamete suspension for this species. Conversely, at lower densities, these indicators of gametic mixing remained notably limited and relatively stable, and the number of isolated colonies increased. These results suggest a lower density range below which effective gametic and, consequently, genetic mixing is limited.

Although theoretical models assessing the retention of genetic diversity in ideal scenarios suggest that founder populations can retain a significant portion of source genetic diversity^70^, these often assume random mating (panmixia), minimum reproductive variance, and uniform gene flow across the population in subsequent generations^71,72^. However, the reality of degraded reefs and small outplanted populations in the context of restoration, exacerbated by factors like fertilisation Allee effects, may violate these assumptions. Reduced colony density and subsequent spatial isolation of colonies limit effective gamete exchange among potential reproductive partners, resulting in substantially smaller effective breeding units. This fundamental constraint on effective gametic mixing reduces the effective population size (*Ne*), possibly increasing the risk of inbreeding depression over time, thereby eroding population genetic diversity and constraining adaptive potential^18,73^. Such conditions promote patterns like chaotic genetic patchiness and ‘sweepstakes reproductive success,’ where successful reproduction is highly localised and dominated by a few individuals or patches^74–76^. Integrating evolutionary genetic modelling with these findings may help refine population density thresholds critical for restoration strategies aimed at sustaining diversity and promoting the spread of beneficial traits^77–79^, though validation using pairwise parental distances from natural populations remains necessary.

These findings have implications for coral outplanting and restoration design, emphasising the importance of spatial arrangement and current direction to maximise reproductive success. Clustered outplants of corals will ultimately result in a greater likelihood of fertilisation success; indeed, high fertilisation rates were observed in the central patch of this study. As a general guideline for restoration activities, colonies should be placed within 5 m of each other to maximise fertilisation success. However, even within the central cluster, some individuals did not participate in fertilisation, likely due to spawning asynchrony or because their offspring were not captured in the subsampled larvae. This incomplete reproductive participation, along with lower fertilisation observed in other studies^38^, highlights the need for outplant patches to be sufficiently large (>20 colonies) and dense to accommodate the possibility of some asynchronous spawning, less favourable hydrodynamic conditions, and to account for colony mortality after outplanting. Predicting current direction at the time of spawning remains challenging without site-specific hydrodynamic data, and its variability further complicates predictions. Maintaining a circular or radial outplanting design may help mitigate risk in the absence of such information. Furthermore, recent progress in image-based survey methods is improving our capacity to quantify spatial population patterns in natural systems. These developments should help refine site-specific population thresholds needed for successful fertilisation, which in turn can guide decisions about when restoration intervention is required^80,81^.

Our method of placing colony fragments along the radial lines and nearby positions may reduce, although not entirely prevent, accurate assessment of fertilisation from the most distant colonies in the experiment. Sperm from nearby colonies are more likely to reach and fertilise eggs sooner than those from distant sources, thereby potentially preventing larger intercolonial distances from being assessed. However, the paternity assignments indicated few fertilisation events between radial lines, with the majority of eggs fertilised from sires of the central patch, indicating this method of control was largely successful. Additionally, our design featured a greater number of close neighbour distances, and we acknowledge that our results may therefore have a bias to lower intercolonial distances than occurs in a natural population and applying paternity assignment approaches to natural reef colony configurations may be an area for future exploration. Yet, recent work by Mumby, et al.^9^ indicated that pairwise nearest neighbour (or intercolonial) distances best described observed fertilisation trends across a natural population in Palau. Other studies have investigated relationships using proxies for adult densities^10,36^. However, density-fertilisation relationships are not always consistent across depths, species, and reproductive modes^37^. Mumby, et al.^9^ highlight the challenges with using ‘density’ as solely a population parameter to assess Allee effects, since clustering can cause density estimates to vary depending on the chosen spatial scale. These results are consistent with Levitan, et al.^82^, who found that larger population sizes of the sea urchin *Strongylocentrotus franciscanus* led to higher fertilisation success, even when density was maintained constant. However, when area was controlled and density was allowed to vary, fertilisation success was influenced by changes in density. Spatially explicit simulation models have supported the use of population density as a relevant population parameter^12,38^, likely because data are spatially averaged to the cell size inflating fine-scale mixing, and unlikely to result in very high or low fertilisation patchiness that nearest-neighbour parameters would determine. Ultimately, it is unlikely that a single population parameter or experimental design on Allee effects during fertilisation can address all these aspects, and multiple approaches combining survey, experimental and modelling exercises are needed^48^.

Our study provides empirical evidence that fine-scale spatial arrangements of adult colonies on reefs are critical for maintaining reproductive success in broadcast spawning corals. As reefs continue to degrade, resulting in lower adult densities and altered spatial clustering, populations will increasingly approach or even exceed the minimum spatial thresholds for effective gametic mixing. These factors are likely to act alongside other processes that can reduce reproductive output^83^. Our results suggest that small, isolated patches of corals with low densities of colonies may face significant Allee effects, ultimately impeding natural recovery and resilience. These findings have implications for restoration strategies: efforts must prioritise not only increasing colony numbers but also ensuring optimal spatial configurations in spawning events to ensure fertilisation success and sustain genetic diversity. Integrating localised hydrodynamic data and tailored outplant designs may therefore be key to mitigating the cascading effects of reef degradation and securing the long-term viability of coral populations.

## Supplementary information

Supplementary Information

Reporting Summary

## Data Availability

The raw genetic sequencing data are available in the NCBI Sequence Read Archive under BioProject accession PRJNA1299555. The datasets used for this study are deposited at https://doi.org/10.6084/m9.figshare.31565230^84^.

## Code availability

The code used for data analysis and modelling described in this study is available at https://doi.org/10.6084/m9.figshare.31649299^85^.
