# Supplementary material for: Proximity and current alignment drive fertilisation success in a broadcast-spawning coral (Acropora cf. hyacinthus)
Source: Commun Biol. 2026 May 9;9:1200. doi: 10.1038/s42003-026-10055-9 (PMC13578429; doi:10.1038/s42003-026-10055-9)
Supplement: Supplementary file 1 [file 42003_2026_10055_MOESM1_ESM.pdf]

Supplementary Material

Note S1. PCA revealed no clear separation of individuals into groups, with the first two principal components explaining only 7.6% and 6.9% of the total variance, respectively (Fig. S1). K-means clustering did not identify distinct groups, and the Elbow method indicated no strong inflection point, suggesting continuous genetic variation rather than discrete clusters. A PERMANOVA test revealed a significant difference between groups at K = 2 ($p < 0.001$), but the effect size was low ($R^2 = 0.067$), suggesting that although some weak genetic differentiation exists, it explains only a small proportion of the overall variance. While ΔK was greatest at K = 2, STRUCTURE analysis exhibited low overall admixture, with most individuals predominantly assigned to a single cluster in the admixture model (data not shown). Mean LnP(K) values decreased slightly from K = 1 to K = 2, indicating that adding an additional cluster did not improve model fit, further supporting a lack of strong genetic structuring. Hardy-Weinberg equilibrium (HWE) tests revealed no loci remained significantly out of equilibrium after FDR correction, consistent with random mating and a panmictic population structure. Collectively, these results indicate weak genetic structuring, if any, with likely high connectivity across this small spatial scale leading to predominantly continuous genetic variation rather than discrete genetic groups.

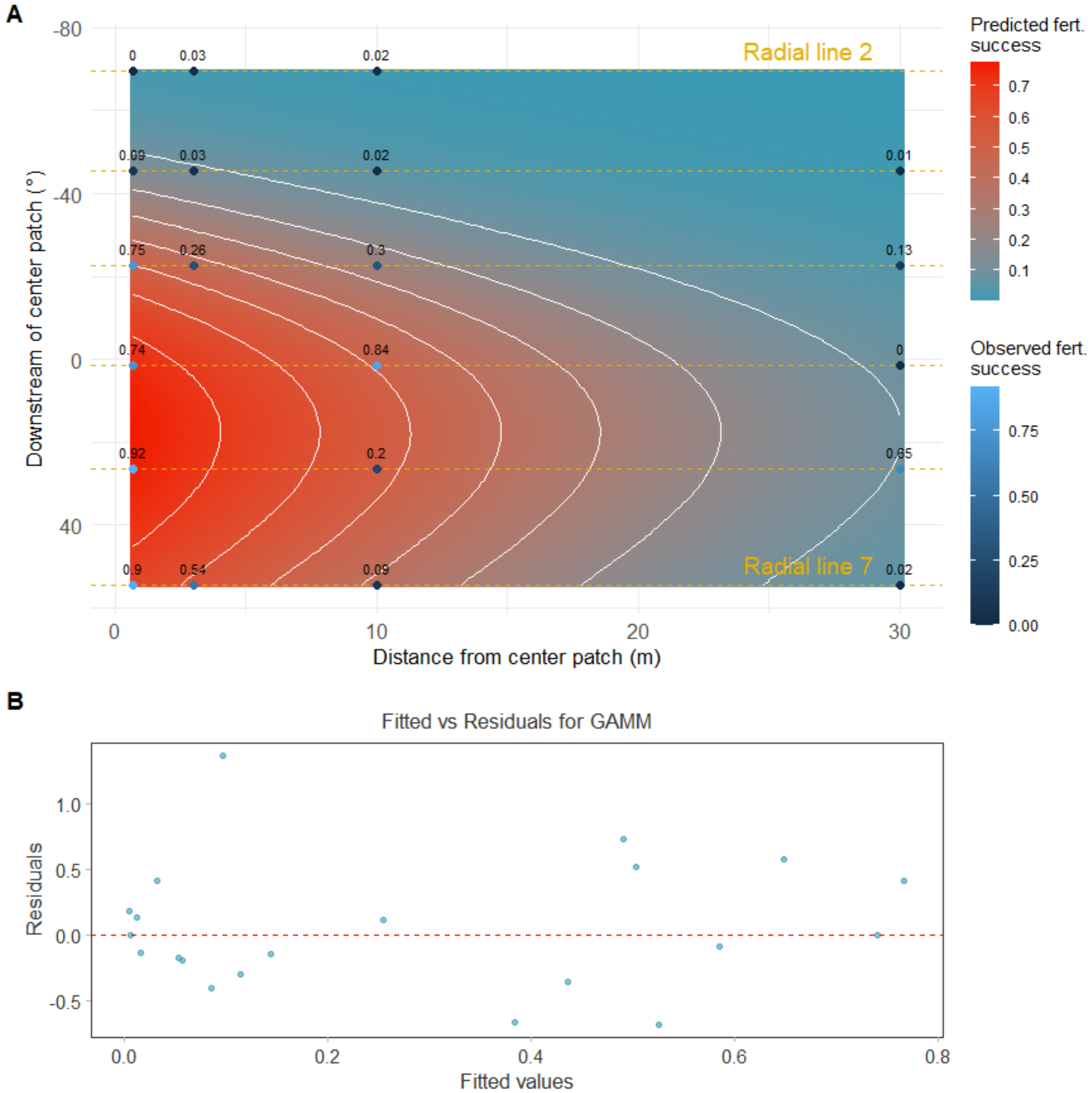

Fig. S1. Model diagnostics for the Generalised Additive Mixed Model (GAMM) resulting from the bulk fertilisation success (proportion) analysis. (A) Observed and predicted fertilisation success for colonies along the radial lines. (B) Fitted vs residuals for the model.

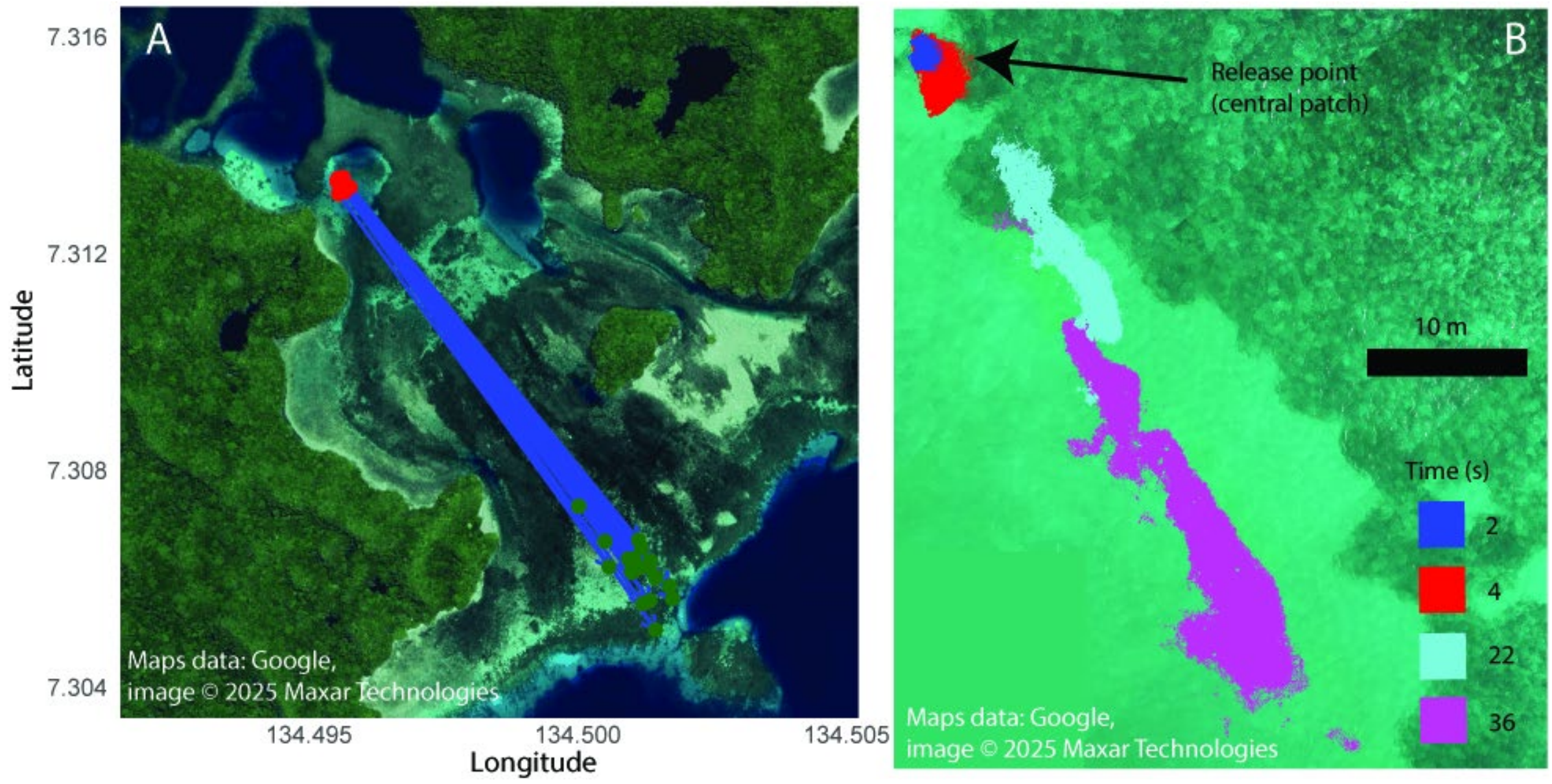

Fig. S2. (A) Trajectories of the experimental containers during the experiment. Red points denote the release locations and green points denote the collection locations. Map data from Google, image © 2025 Maxar Technologies. (B) Movement of a tracer dye release over time (s) from the central patch, recorded during an ebb tidal flow comparable to that of the field experiment.

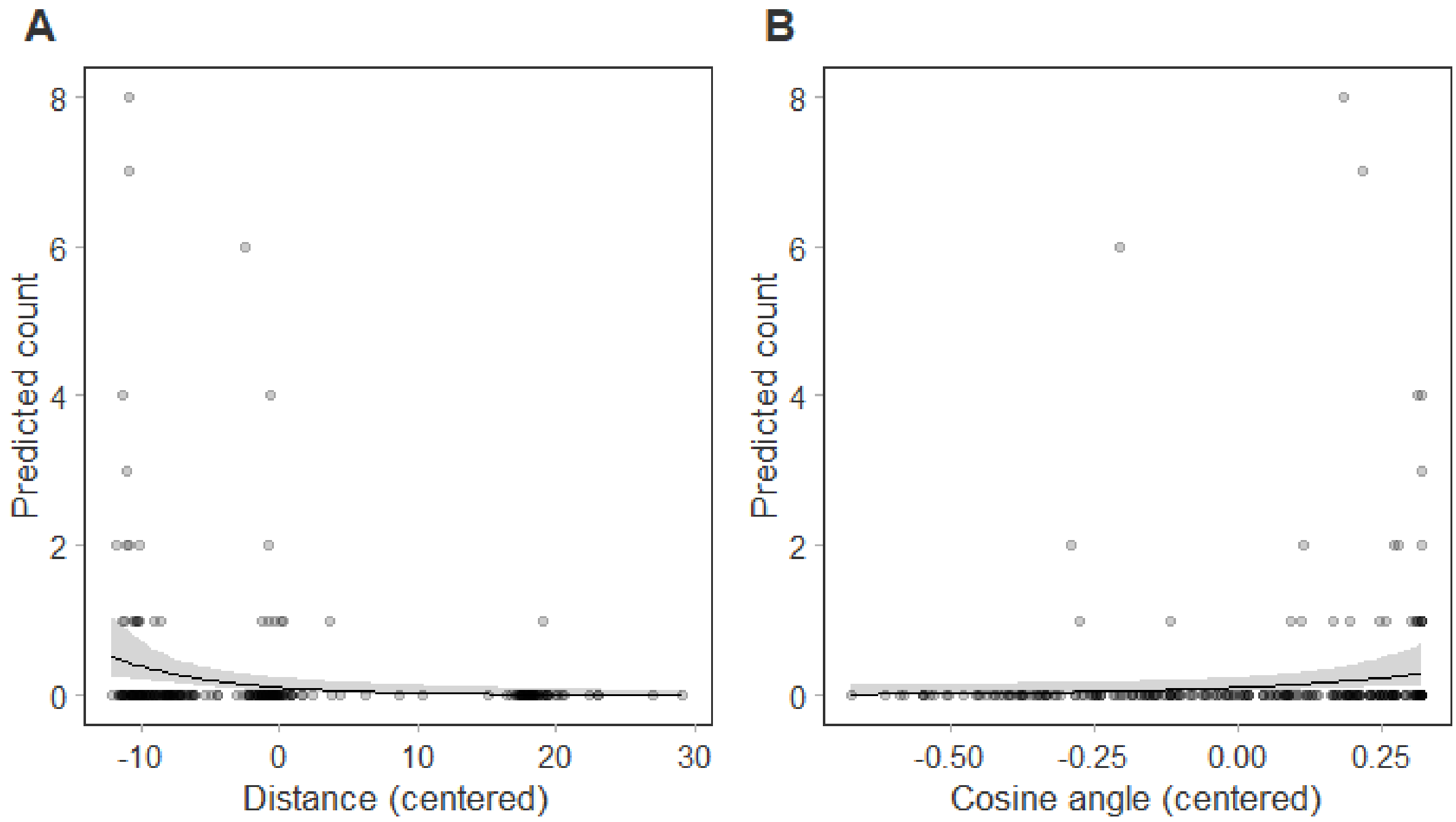

Fig. S3. Effect plots of the predicted relationships between assigned parents and explanatory variables. Panels show the effects of (A) physical distance between colonies, (B) relative angular position of colonies to upstream/downstream angle, while holding other covariates at their mean values. Points represent raw data, solid lines show model predictions, and shaded areas represent 95% confidence intervals. Note that distance and angle values are mean-centred.

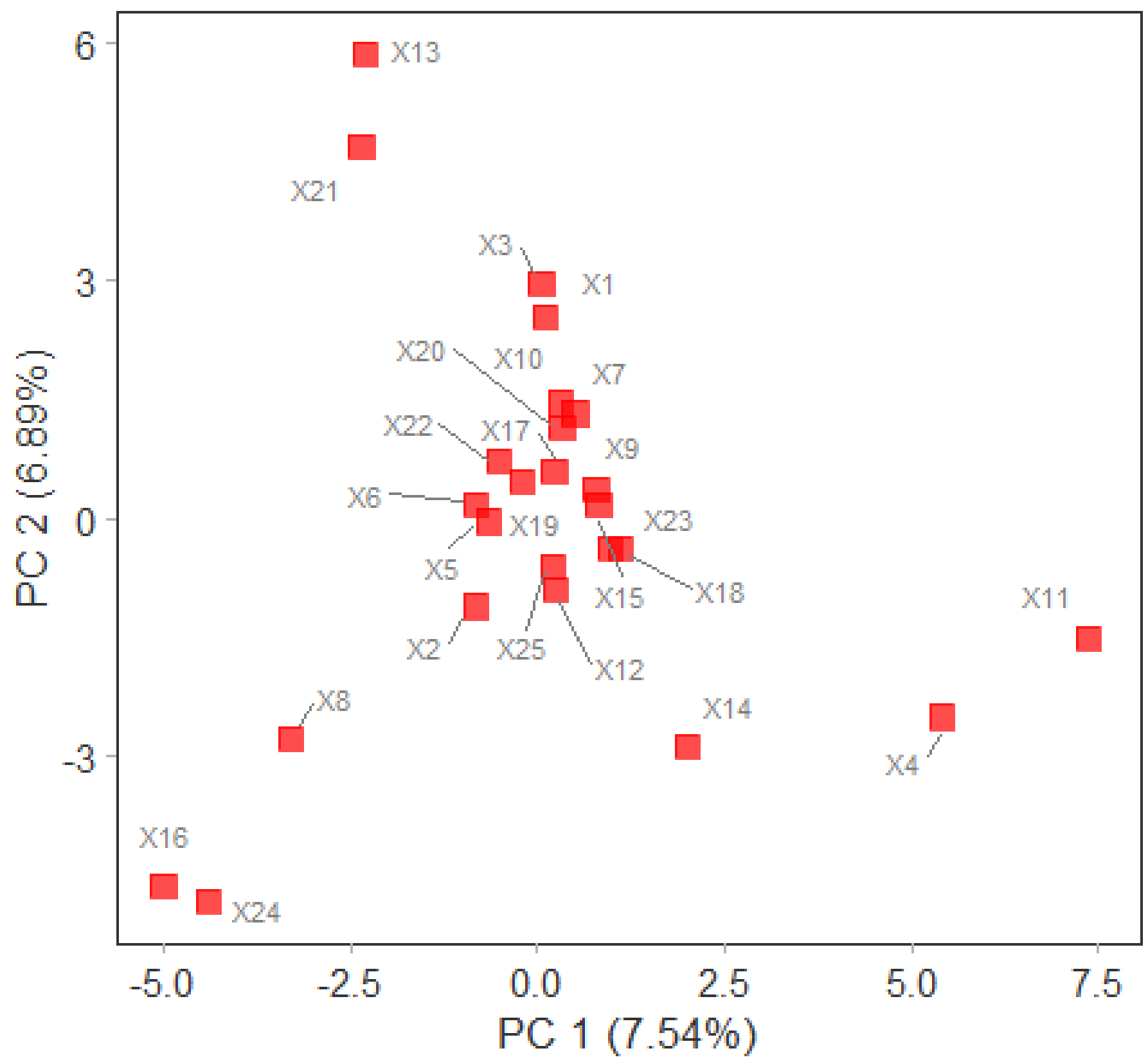

Fig. S4. Principal Component Analysis (PCA) plot of distinct adult colonies of Acropora cf. hyacinthus, showing ordination along the first two principal components.

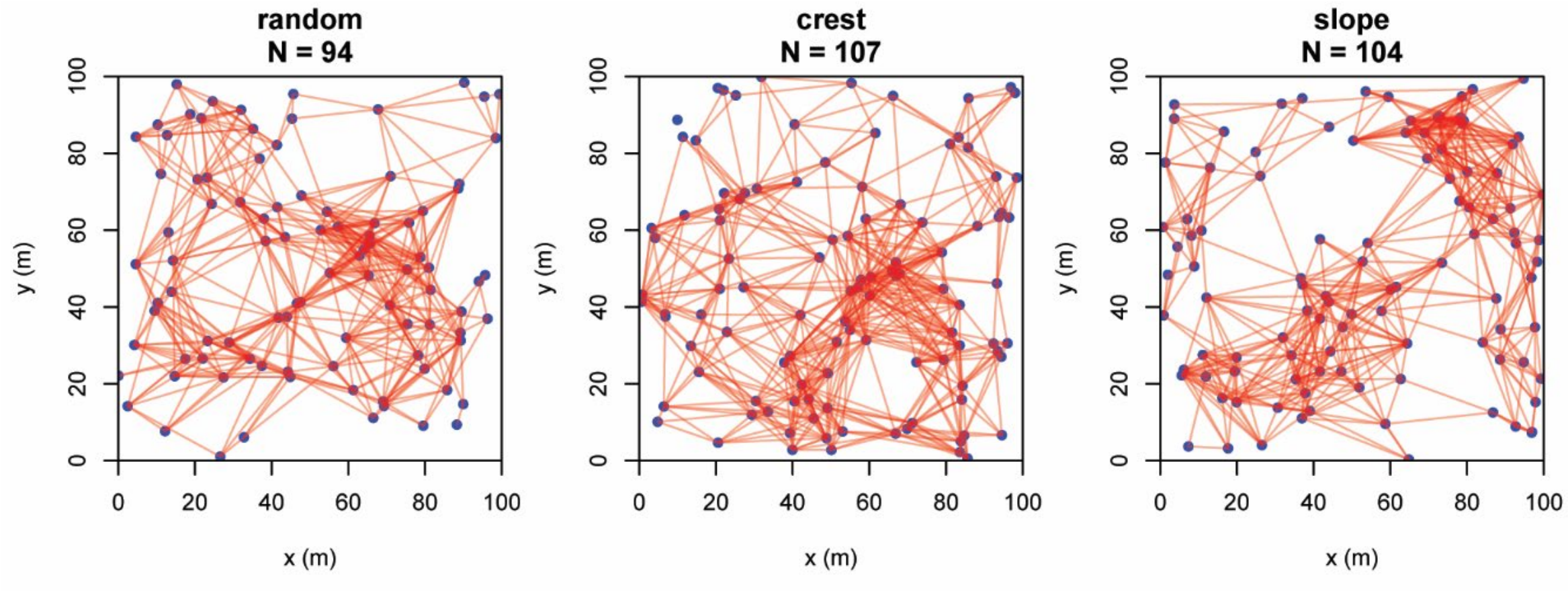

Fig. S5. A representative example of simulated pairwise crosses at each reef spatial distribution (reef slope, random/Poisson, and reef crest) for the density = 0.01 colonies m^{-2} (100 colonies per hectare) with a greater number of connections aligned west–east, consistent with the direction of the prevailing current. Note that population size varies slightly between each run due to the random spatial arrangement.
