# Supplementary material for: Proximity and current alignment drive fertilisation success in a broadcast-spawning coral (Acropora cf. hyacinthus)
Source: Commun Biol. 2026 May 9;9:1200. doi: 10.1038/s42003-026-10055-9 (PMC13578429; doi:10.1038/s42003-026-10055-9)
Supplement: Supplementary file 2 [file 42003_2026_10055_MOESM2_ESM.pdf]

nature portfolio

Corresponding author(s): Gerard Ricardo

Last updated by author(s): Aug 12, 2025

Reporting Summary

Nature Portfolio wishes to improve the reproducibility of the work that we publish. This form provides structure for consistency and transparency in reporting. For further information on Nature Portfolio policies, see our Editorial Policies and the Editorial Policy Checklist.

Statistics

For all statistical analyses, confirm that the following items are present in the figure legend, table legend, main text, or Methods section.

n/a	Confirmed	
☐	☒	The exact sample size (*n*) for each experimental group/condition, given as a discrete number and unit of measurement
☐	☒	A statement on whether measurements were taken from distinct samples or whether the same sample was measured repeatedly
☐	☒	The statistical test(s) used AND whether they are one- or two-sided<br>*Only common tests should be described solely by name; describe more complex techniques in the Methods section.*
☐	☒	A description of all covariates tested
☐	☒	A description of any assumptions or corrections, such as tests of normality and adjustment for multiple comparisons
☐	☒	A full description of the statistical parameters including central tendency (e.g. means) or other basic estimates (e.g. regression coefficient) AND variation (e.g. standard deviation) or associated estimates of uncertainty (e.g. confidence intervals)
☐	☒	For null hypothesis testing, the test statistic (e.g. *F*, *t*, *r*) with confidence intervals, effect sizes, degrees of freedom and *P* value noted<br>*Give P values as exact values whenever suitable.*
☒	☐	For Bayesian analysis, information on the choice of priors and Markov chain Monte Carlo settings
☐	☒	For hierarchical and complex designs, identification of the appropriate level for tests and full reporting of outcomes
☒	☐	Estimates of effect sizes (e.g. Cohen's *d*, Pearson's *r*), indicating how they were calculated

Our web collection on statistics for biologists contains articles on many of the points above.

Software and code

Policy information about availability of computer code

Data collection	No software was used for data collection.
Data analysis	R (v. = >4.4.1), CERVUS (3.0.7) and COLONY (2.0.7.1) were used to analyse the data.

For manuscripts utilizing custom algorithms or software that are central to the research but not yet described in published literature, software must be made available to editors and reviewers. We strongly encourage code deposition in a community repository (e.g. GitHub). See the Nature Portfolio guidelines for submitting code & software for further information.

Data

Policy information about availability of data

All manuscripts must include a data availability statement. This statement should provide the following information, where applicable:

- Accession codes, unique identifiers, or web links for publicly available datasets
- A description of any restrictions on data availability
- For clinical datasets or third party data, please ensure that the statement adheres to our policy

TThe raw genetic sequencing data are available in the NCBI Sequence Read Archive under BioProject accession PRJNA1299555. Details for the source data and code are provided within the manuscript.

Research involving human participants, their data, or biological material

Policy information about studies with human participants or human data. See also policy information about sex, gender (identity/presentation), and sexual orientation and race, ethnicity and racism.

Reporting on sex and gender	NA
Reporting on race, ethnicity, or other socially relevant groupings	NA
Population characteristics	NA
Recruitment	NA
Ethics oversight	NA

Note that full information on the approval of the study protocol must also be provided in the manuscript.

Field-specific reporting

Please select the one below that is the best fit for your research. If you are not sure, read the appropriate sections before making your selection.

☐ Life sciences ☐ Behavioural & social sciences ☒ Ecological, evolutionary & environmental sciences

For a reference copy of the document with all sections, see nature.com/documents/nr-reporting-summary-flat.pdf

Ecological, evolutionary & environmental sciences study design

All studies must disclose on these points even when the disclosure is negative.

Study description	This study examined the fertilisation success of the reef-building table coral Acropora cf. hyacinthus in Palau, Micronesia. The research aimed to assess the effect of intercolonial spacing and spatial arrangement (position relative to the current direction) of spawning colonies on fertilisation success using a manipulated field experiment. The experimental design involved a central high-density patch of gravid corals (n = 17 colonies) with multiple radial lines extending outward, each containing 4 colonies spaced at intervals. Mesh containers holding eggs from designated donor colonies on the radial lines and the central patch (n = 28) were released into the gamete plume to assess fertilisation success, with paternity assignments performed on the resulting embryos. Each mesh container typically contained >200 eggs prior to release.
Research sample	The study focused on a single species, Acropora cf. hyacinthus. Twenty-five medium-sized, genetically distinct colonies were collected from the offshore Uchelbeluu Reef. This species was selected due to its fast growth and role in reef recovery. Individuals assigned to radial lines were fragmented along these lines to prevent cross-fertilisation between nearby individuals biasing results.
Sampling strategy	A total of 25 of 28 containers were successfully retrieved. Due to the potential for nearby colonies to bias distance–dependent relationships, only one colony was positioned at each distance along a given radial line. Although replication at identical distances was not possible, we assessed six radial lines oriented at different angles to the current direction and treated colony position as a continuous spatial variable in our regression analysis, allowing all observations to contribute to parameter estimation, thereby reducing the need for strict distance–based replication. A simple sample size simulation was performed to detect a 10% decrease in fertilisation success over 30 metres, ignoring current direction effects. Assuming an average of 200 eggs per container and 80% fertilisation success at 0 metres, the required total number of colonies to achieve $\beta = 0.8$ was estimated at 19 along all radial lines; the study included 24 colonies. No formal sample size calculation was performed for the paternity assignments due to biological and technical uncertainties, including variable fertilisation success, DNA extraction efficiency and sequencing quality.
Data collection	A team of seven divers released the mesh containers from the designated egg donor colonies during the field experiment. Afterwards, a team of five researchers microscopically assessed the eggs to evaluate fertilisation success and isolate embryos for genetic sequencing.
Timing and spatial scale	The manipulated field experiment was conducted during the annual coral spawning season in Palau on April 2nd, 2023. The experiment was carried out at night over several hours. Fertilisation was tracked for approximately 80–90 minutes after the release of the mesh containers. Colony fecundity was assessed the following day and <5% of the branches still contained eggs, preventing a second experiment.
Data exclusions	One of the self-fertilisation controls was disregarded due to suspected contamination, as it showed an unrealistically high fertilisation rate (18.1%). Additionally, Paternity assignments where sires could not be distinguished between multiple spatial positions in the experimental design were not included in the analysis.
Reproducibility	The opportunistic and time-constrained nature of the experiment prevents direct replication, as metocean conditions change. Fertilisation assessments were conducted using well-established methods.

Randomization	Colonies were randomly placed within the central patch to produce the sperm cloud. Colonies that were fragmented along the radial lines were likewise randomly selected.
Blinding	Blinding was not feasible due to the nature of the field experiments, in which divers were actively involved in placing and monitoring coral colonies, as well as tracking the containers during the fertilisation process.

Did the study involve field work? ☒ Yes ☐ No

Field work, collection and transport

Field conditions	The spawning in the area coincided with warm doldrum conditions likely resulting in nearshore coral spawning earlier than predicted. On the night of the experiment, the tide was at mid-ebb (0.67 amplitude), flowing SSE toward the bay entrance. The site was largely sheltered from the prevailing 2.4-knot NNW offshore wind. Water depth downstream of the experimental setup varied between 1 and 3 m, with mesh containers drifting at a mean velocity of 0.21 m s-1.
Location	The experiment was conducted at Ngermid Bay in Palau (7°18'54"N, 134°29'42"E), where coral patches were positioned at depths of 2–3 metres in a semi-enclosed bay protected from prevailing winds by surrounding rock islands. The location was selected for its hydrodynamic features, isolating the experimental coral patches from wild populations to minimise contamination from external gametes.
Access & import/export	The work was conducted under CITES permit PW23-079 and PWS2023-AU-001443, Bureau of Marine Resources #100939-E, and Marine Research Permit RE-22-11.
Disturbance	Adult colonies were returned to their original collection site following spawning.

Reporting for specific materials, systems and methods

We require information from authors about some types of materials, experimental systems and methods used in many studies. Here, indicate whether each material, system or method listed is relevant to your study. If you are not sure if a list item applies to your research, read the appropriate section before selecting a response.

Materials & experimental systems

n/a	Involved in the study	
☒	☐	Antibodies
☒	☐	Eukaryotic cell lines
☒	☐	Palaeontology and archaeology
☐	☒	Animals and other organisms
☒	☐	Clinical data
☒	☐	Dual use research of concern
☒	☐	Plants

Methods

n/a	Involved in the study	
☒	☐	ChIP-seq
☒	☐	Flow cytometry
☒	☐	MRI-based neuroimaging

Animals and other research organisms

Policy information about studies involving animals; ARRIVE guidelines recommended for reporting animal research, and Sex and Gender in Research

Laboratory animals	The study did not use laboratory animals
Wild animals	All corals were returned to their original collection site. The colonies were removed from the substrate using standard removal practices i.e., a hammer and chisel to break free the base of their skeleton. The colonies were transported submerged in large tubs, and positioned into the experimental patch, and elevated off the substrate to prevent sediment scour.
Reporting on sex	The corals used here are hermaphroditic and bundle their eggs and sperm together during release.
Field-collected samples	This study did not include field-collected samples housed in aquaria.
Ethics oversight	No ethics oversight was required for this study.

Note that full information on the approval of the study protocol must also be provided in the manuscript.

Plants

Seed stocks	NA
Novel plant genotypes	NA
Authentication	NA
